# EUS-Guided Biliary Drainage

**DOI:** 10.1155/2012/348719

**Published:** 2011-08-14

**Authors:** Marc Giovannini, Erwan Bories

**Affiliations:** Endoscopic Unit, Paoli-Calmettes Institute, 232 Boulevard St-Marguerite, 13273 Marseille Cedex 9, France

## Abstract

The echoendoscopic biliary drainage is an option to treat obstructive jaundices when ERCP drainage fails. These procedures compose alternative methods to the side of surgery and percutaneous transhepatic biliary drainage, and it was only possible by the continuous development and improvement of echoendoscopes and accessories. The development of linear setorial array echoendoscopes in early 1990 brought a new approach to diagnostic and therapeutic dimenion on echoendoscopy capabilities, opening the possibility to perform punction over direct ultrasonographic view. Despite of the high success rate and low morbidity of biliary drainage obtained by ERCP, difficulty could be found at the presence of stent tumor ingrown, tumor gut compression, periampulary diverticula, and anatomic variation. The echoendoscopic technique starts performing punction and contrast of the left biliary tree. When performed from gastric wall, the access is made through hepatic segment III. From duodenum, direct common bile duct punction. Dilatation is required before stent introduction, and a plastic or metallic stent is introduced. This phrase should be replaced by: diathermic dilatation of the puncturing tract is required using a 6F cystostome. The technical success of hepaticogastrostomy is near 98%, and complications are present in 36%: pneumoperitoneum, choleperitoneum, infection, and stent disfunction. To prevent bile leakage, we have used the 2 stent techniques, the first stent introduced was a long uncovered metallic stent (8 or 10 cm), and inside this first stent a second fully covered stent of 6 cm was delivered to bridge the bile duct and the stomach. Choledochoduodenostomy overall success rate is 92% and described complications include, in frequency order, pneumoperitoneum and focal bile peritonitis, present in 19%. By the last 10 years, the technique was especially performed in reference centers, by ERCP experienced groups, and this seems to be a general guideline to safer procedure execution.

## 1. Introduction

Endoscopic biliary stenting is the most common method to treat obstructive jaundice. But in 3–12% of cases, selective cannulation of the major papilla failed and surgery or percutaneous biliary drainage is required. But percutaneous drainage needed dilated intrahepatic biliary ducts and the rate of complications reaches 25–30% of cases including peritoneal bleeding. A new technique of biliary drainage using EUS and EUS-guided puncture of the bile duct (common bile duct or left hepatic duct) is now possible. 

 Using EUS guidance and dedicated accessories it's now possible to create biliodigestive anastomosis. 

The aim of this paper is:

1. to describe the material needed for such procedures,
2. to describe the technique of biliary drainage under EUS guidance,
3. to describe the place today of these techniques in comparison with ERCP.

## 2. Material

### 2.1. Interventional Echoendoscopes

Around 1990, the Pentax-Corporation developed an electronic convex curved linear array echoendoscope (FG32UA) with an imaging plane in the long axis of the device that overlaps with the instrumentation plane. This echoendoscope, equipped with a 2.0 mm working channel, enabled fine-needle biopsy under EUS guidance. However, the relatively small working channel of the FG 32UA was a drawback for pseudocyst drainage since it necessitated the exchange of the echoendoscope for a therapeutic duodenoscope to insert either a stent or nasocystic drain. To enable stent placement using an echoendoscope, the EUS interventional echoendoscopes (FG 38X, EG 38UT, and EG 3870UTK) were developed by Pentax-Hitachi. The FG 38X has a working channel of 3.2 mm, which allows the insertion of a 8.5F stent or nasocystic drain and the EG38UT and EG3870UTK have a larger working channel of 3.8 mm with an elevator allowing the placement of a 10F stent [1, 2]. 

The Olympus Corporation has also developed convex array echoendoscopes. The GF UC 30P has a biopsy channel of 2.8 mm, which enables the placement of a 7-french stent or nasocystic catheter, and the instrument is equipped with an elevator. A new prototype, the GF UCT 30, has a larger working-channel of 3.7 mm allowing the placement of 10-french stent. The main drawback of convex linear array echoendoscopes is the more limited imaging field (120° using the Pentax and 180° using the Olympus) produced by an electronic transducer. These instruments are coupled with the Aloka processor or with a smaller processor (Suzie).

### 2.2. Needles and Accessories for Drainage

**Figure 1:**
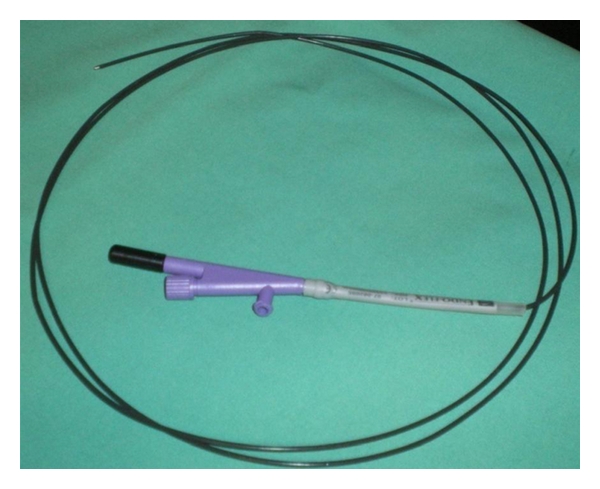
6F cystostome (Endoflex company).

Some authors have used needle knife catheters, but the needle can be difficult to visualize endosonographically. The “Zimmon” needle-knife (Wilson-Cook Corporation, Winston Salem, NC, USA) has a large gauge needle that is easier to visualize. Diathermy is usually required to penetrate the cyst [3] (Figure 1).

**Figure 2:**
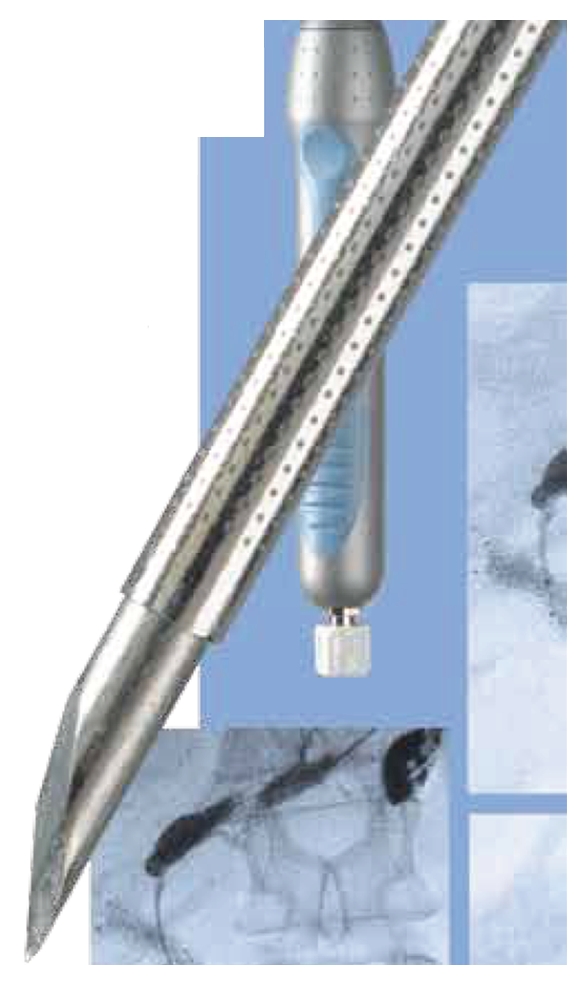
Echotip “ACCESS NEEDLE” Cook company.

A standard endosonography fine needle aspiration (FNA) needle is well visualized sonographically and can be used for pseudocyst puncture. The drawback of this needle is the small caliber (22 or 23 G) that will accept only a 0.018-inch guidewire. Using a 19 G FNA needle (Wilson-Cook Corporation), a 0.0035-inch guidewire can be inserted through the needle into the dilated bile duct. Wilson Cook Corporation has recently developed a “new access needle”; However, one of the main problems during these new techniques of hepaticogastrostomy, is the difficulty manipulating the wire guide through the 19-gauge EUS needle. The main trouble was the “stripping” of the coating of the wire, which in turn created a risk of leaving a part of the wire coating in the patient and also the impossibility to continue the procedure and to insert the stent. To solve this problem, we worked with Cook Medical to design a special needle called the EchoTip Access Needle* (Figure 2). This needle is original because the stylet is sharp and it is relatively easy to insert the needle into the bile duct or the pancreatic duct or a pseudocyst. When the stylet is withdrawn, the needle left in place is smooth and the manipulation of the wire guide is easy and the device is designed to decrease the possibility of the wire stripping.

## 3. EUS-Guided Rendez-Vous Technique

After puncture of the left hepatic biliary system (see above) using a 19-gauges needle (Echo−1−19; Cook Endoscopy), a 0.035-inch hydrophilic guidewire (Tracer Metro Direct, Cook Endoscopy or Jagwire, Boston scientific, Paris, France) was inserted into the biliary duct and then rolled up inside the duodenum. Then, echoendoscope was gently withdrawn leaving the guidewire in place. Afterwards, a duodenoscope was inserted in parallel of the guidewire and placed in the third duodenum, allowing retrograde approach. Guidewire was then catched with standard snare through the working channel and after over-the-wire biliary sphincteromy, stones removal or stent placement could be achieve as usually.

## 4. EUS-Choledocoduodenostomy

A 19-G needle (EchoTip; Wilson-Cook) is inserted trans-duodenally into the bile duct under EUS guidance. Bile is aspirated and contrast medium is injected into the bile duct for cholangiography. A 450-cm long, 0.035-inch guidewire is inserted into through the 19-G needle into the bile duct. The choledochoduodenal fistula is dilated using a biliary catheter for dilation (Soehendra biliary dilator; Wilson-Cook), or a 6F cystostome (Endoflex, company). A 7 Fr to 10 Fr biliary plastic stent or a covered self-expandable metallic stent is placed through the choledochoduodenostomy site into the extrahepatic bile duct.

## 5. Technique of Left Hepaticogastrostomy under EUS Guidance (HGE) (Figure 3)

EUS-guided hepaticogastrostomy was first reported by Burmester [4] in 2003. The technique is also basically similar to EUS-guided drainage of pancreatic pseudocysts. By using an interventional echoendoscope, the dilated left hepatic duct (segment III) was well visualized. HGE was then performed under combined fluoroscopic and ultrasound guidance, with the tip of the echoendoscope positioned such that the inflated balloon was in the middle part of the small curvature of the stomach. A needle (19 G, EchoTip Access Needle, Cook Ireland Ltd., Limerick, Ireland) was inserted transgastrically into the distal part of the left hepatic duct and contrast medium was injected. Opacification demonstrated a dilated biliary ducts to the complete obstruction. The needle was exchanged over a guidewire (0.02-inch diameter, Terumo Europe, Leuven, Belgium) for a 6.5F diathermic sheath (prototype Cysto-Gastro set, EndoFlex, Voerde, Germany), which was then used to enlarge the channel between the stomach and the left hepatic duct. The sheath was introduced by using cutting current. After exchange over a guidewire (TFE-coated 0.035-inch diameter, Cook Europe, Bjaeverskov, Denmark), a 8.5F, 8-cm—long hepatico-gastric stent) or a covered metallic expandable stent (Boston-scientific, 8 cm length) was positioned.As observed by fluoroscopy, contrast emptied from the stent into the stomach. To prevent bile leakage you can leave through the metallic stent a 6 or 7F naso-biliary drain in aspiration during 48 hours. More recently we decided to combine an uncovered stent and a covered stent inserted into. Among these, hepaticogastrostomy was sometimes combined with placement of an additional metallic stent bridging the distal stricture.

## 6. Place of the Bilio-Digestive Anastomosis Guided by EUS in Comparison with ERCP

ERCP is still today the Gold Standard technique for the drainage of an obstructive jaundice due to a pancreatic cancer. Success rate of biliary stenting using ERCP is around 80–85% but sometime ERCP failed to cannulate selectively the papilla or failed to reach the papilla in case of duodenal obstruction. These new techniques of biliary drainage using EUS guidance could be an alternative to percutaneous procedures or to Surgery. 

The problem with the percutaneous techniques of biliary drainage is the high rate of complication (bleeding, peritoneal bile leakage) around 20–30% of the cases and the morbidity and the mortality of Surgery for such palliative procedures are, respectively, of 35–50% and 10–15%.

For probably, these new techniques of biliary drainages will be in the future an alternative to Surgery and percutaneous biliary drainage. 

 To date, 120 patients with EUS-guided bile duct drainage have been reported in thirteen studies (Table 1). 19-gauge or 22-gauge fine needles or fine needles followed by needle knife or cystotome were used for puncturing intrahepatic bile ducts in all of the patients. Hepaticogastrostomy was successful in all but two cases (49/51, 96%). Various types of stents, including plastic stents, uncovered MS, and covered MS were used for the drainage. Once the stents were placed, all but one patient (48/49, 98%) had successful resolution of obstructive jaundice. The rate of procedurerelated early complications was 19% (5 mild and 5 severe) with one death: 1 case of ileus probably due to the use of morphine during anesthesia, 1 case of bilioma, and 2 cases of cholangitis. Stent migration has been reported as a late complication in one case. Kahaleh et al. described that the advantages of EUS-guided hepaticogastrostomy over percutaneous transhepatic drainage included puncture of the biliary tree with real-time US when using color-Doppler information to limit the possibility of vascular injury, the lack of ascites in the interventional field when present in the peritoneum, and the lack of an external drain. And based on their experience, they also pointed out the extrahepatic approach has a greater chance of complication than the intrahepatic approach. Itoi et al. reported the limitations of this technique as follows, (i) nonapposed gastric wall and the left liver lobe, with a certain displacement between the puncture site of the gastric wall and intrahepatic bile duct, resulting in possibility of procedure failure. (ii) risk of mediastinitis with a transesophageal approach, (iii) difficulty of puncture in case of liver cirrhosis, (iv) risk of injuring the portal vein and (v) necessitating the use of small-caliber stents or MS with a small-diameter delivery device [17].

From a clinical standpoint, however, the most relevant technical choice appears to be the type of stent. As detailed in Table 1, 7 to 8.5 plastic stents were placed in 46% of cases, whereas uncovered, partially covered or fully covered SEMS were placed initially in 54%. It is difficult to draw significant conclusions from the published reports, since no formal comparisons have been made between the two types of stents. SEMS are appealing for three reasons. First, upon full expansion SEMS effectively seal the puncture/dilation tract, which would in theory prevent leakage. Secondly, their larger diameter provides better long-term patency, which would decrease the need for stent revisions. Finally, if dysfunction by ingrowth or clogging occurs, management is somewhat less challenging than with plastic stents, since a new stent (plastic or SEMS) can easily be inserted through the occluded SEMS in place. In contrast, exchanging a clogged plastic transmural stent usually requires over-the-wire replacement, because free-hand removal involves the risk of track disruption with subsequent guidewire passage into the peritoneum, hence requiring repeat EUSBD if drainage is to be reestablished [18]. These presumed advantages of SEMS must be balanced against the fact that transmural SEMS insertion and deployment are somewhat more demanding than they are at ERCP. In particular, the serious risk of foreshortening and bile peritonitis should be prevented with careful attention to details [15].

We reported recently our experience on 38 patients [19] (F = 20, Mean age = 66.5 yrs, (38–93 yrs)) were referred for management of biliary disorders: benign disease in 11 (iatrogenic stenosis = 8, chronic pancreatitis = 1, fistula = 1, bile duct dilation = 1) and malignant in 27 (pancreatic cancer = 10, cholangiocarcinoma = 10, other = 7). EUS approach was chosen after failure of ERCP (*n* = 9), impossibility to reach papilla (duodenal strictures = 6, postsurgical anatomy = 9) or incomplete left bile duct drainage (*n* = 14). All procedures were realized using therapeutic echoendoscope, and fluoroscopic guidance. EUS procedures were performed using transgastric approach. Stents were placed transpapillary (transpapillary stent insertion), between the stomach and the left liver lobe to keep the fistula open (hepaticogastrostomy) or both. 41 EUS-guided biliary procedures were realized. Choleperitoneum occurred in 1 casen treated medically. 36 transgastric approaches were performed in 35 patients with technical success in 97%. All stents placed under EUS guidance were clinically efficient. Complications occurred in 25% (*n* = 9, choleperitoneum = 5, stent migration = 3, liver abscess = 1). All complications were managed conservatively. 1 patient died secondary to severe choleperitoneum. 

## 7. Conclusion

EUS-guided biliary management is useful in case of failure of ERCP with a high rate of technical success and clinical efficacy. Morbidity rate is high during biliary drainage requiring experienced team. In summary. EUS-guided biliary procedure open a new way to achieve biliary drainage, complementary to percutaneous approach. Hepaticogastrostomy is feasible providing high success rate. Nevertheless morbidity rate is still elevated. Further technical improvements are therefore mandatory to reduces a number of adverse events.

## Figures and Tables

**Figure 3:**
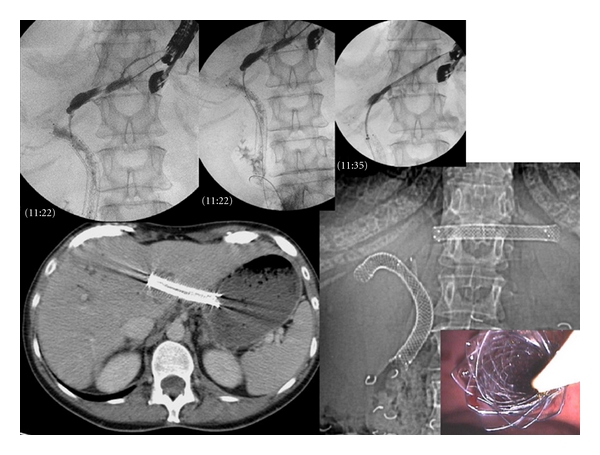
Hepaticogastrostomy performed after ERCP failed to drain the left hepatic lobe in patient with a Klatskin Tumor.

**Table 1:** Summary of the published literature on EUS-HG and related transmural intrahepatic EUSBD.

Author/year/ref	*n* Total	*n* IH-transmural		Success		Complications		Initial stent	
	EUSBD	EUSHG nonHG		technical	Clinical	*n*	Type	Plastic	SEMS
Burmester et al. [4] 2003	4	1	1	2	2	0	—	2	0
Püspök et al. [5] 2005	6	0	1	1	1	0	—	1	0
Artifon et al. [6] 2007	1	1	0	1	1	0	—	0	1
Bories et al. [7] 2007	11	11	0	10	10	4	2 cholangitis, 1 ileus, 1 biloma	7	3
Will et al. [8] 2007	8	4	4	7	6	2	1 cholangitis, 1 pain	2	5
Chopin-Laly et al. [9] 2004	1	1	0	1	1	0	—	0	1
Iglesias-García et al. [10] 2008	1	1	0	1	1	0	—	NS	NS
Horaguchi et al. [11] 2009	16	5	2	7	6	1	Cholangitis	7	0
Maranki et al. [12] 2009	49	3	0	3	3	0	—	3	0
Park et al. [13] 2009	14	8	1	9	9	2	Pneumo	0	9
Park et al. [14] 2010	5	5	0	5	5	0	—	0	5
Martins et al. [15] 2010	1	1	0	1	0	1	Peritonitis and death	0	1
Eum et al. [16] 2010	3	1	0	1	1	0	—	0	1

Total	120	42	9	49	46	10	5 mild/5 severe	22	26
